# Gamma Knife radiosurgery for vestibular schwannomas: Evaluation of planning using the sphericity degree of the target volume

**DOI:** 10.1371/journal.pone.0225638

**Published:** 2020-01-10

**Authors:** Crystian Wilian Chagas Saraiva, Simone Coutinho Cardoso, Daniela Piai Groppo, Antônio Afonso Ferreira De Salles, Luiz Fernando de Ávila, Luiz Antonio Ribeiro da Rosa

**Affiliations:** 1 Instituto de Radioproteção e Dosimetria / Comissão Nacional de Energia Nuclear–IRD / CNEN, Rio de Janeiro, Brazil; 2 Associação do Sanatório Sírio—Hospital do Coração–HCor, São Paulo, Brazil; 3 Universidade Federal do Rio de Janeiro–Instituto de Física–UFRJ, Rio de Janeiro, Brazil; 4 Instituto de Pesquisa Energéticas e Nucleares–IPEN / CNEN, São Paulo, Brazil; 5 Universidade Estadual de Campinas, Faculdade de Tecnologia–UNICAMP, Limeira, Brazil; St. Vincent Medical Center, UNITED STATES

## Abstract

**Introduction:**

This study explores the possibility of a relationship between the sphericity degree of a target volume with the dose distribution. This relationship is evaluated based on the ratio isodose volume / target volume (IV/TV) and the metrics coverage, i.e., selectivity, gradient index, conformity index and mean dose when planning radiosurgery for vestibular schwannoma.

**Methods:**

Sphericity degree (*φ*) was calculated for each target volume (TV) of 64 patients who underwent stereotactic radiosurgery (SRS) for vestibular schwannoma. The calculation of this parameter was developed using the theoretical definition for operational sphericity *φ* = *V*_*P*_/*V*_*CS*_. The values found are evaluated considering the following metrics:—Coverage (C), selectivity (S), gradient index (GI), Paddick conformity index (CI_Paddick_) and dose distribution (IV/TV). The planning was also carried out considering a spherical target volume defined in a spherical phantom. The spherical volume is the same as the target used in the treatment plan. The planning of the spherical target was considered as a reference plan to evaluate the dose distribution inside and outside the volume.

**Results:**

It was possible to observe that the majority of target volumes has (ϕ) around 0,66–0,77, corresponding to 54,7% of the total. Considering the mean values for metrics, the results are: C = 0,98, S = 0,78, GI = 3,11 and CI = 0,81. The dose distribution was equivalent for treatment plans and reference plans. Quantitative analysis for IV/TV shows that these values are higher than 30% for treatment plans where shot density is large.

**Conclusion:**

This study demonstrates that de sphericity degree (*φ*) can be related to the dose distribution (IV/TV). Therefore the sphericity degree is a good parameter to evaluate the dose distribution of a plan for vestibular schwannoma treatment, considering the reference plan as being a spherical target using a leksell gamma knife^®^ perfexion (LGKP). This study shows that the sphericity degree offers important information of the dose distribution outside and inside the target volume. This is not evaluated by the other parameters already implemented as metric to analyzing the GKP plans.

## I. Introduction

The vestibular schwannoma is a benign intracranial tumor, also called an acoustic neuroma. The treatment of this tumor may be surgical and/or using radiosurgery. This treatment modality has shown excellent results, for this reason the radiosurgery is much used to treat vestibular schwannoma [1–4], The treatment using radiosurgery consists of a non-invasive treatment using beams of X-rays or gamma rays, both with high energy, to deliver high-dose radiation to the tumor while minimizes the dose to surrounding healthy brain tissue [2–4]. The aim of the radiosurgical treatment of acoustic neuroma is to stop its growth permanently.

As a tool for a radiosurgical treatment, the Leksell *Gamma knife*^*®*^ *Perfexion* (LGKP), (Elekta Instruments, Stockholm, Sweden), is a completely redesigned system that was introduced in 2006. The treatment planning (dose calculation) for radiosurgery of vestibular schwannoma with LGKP equipment is done using a treatment planning system–TPS, called *Leksell Gamma Plan*^*®*^ (LGP), (Elekta Instruments, Stockholm, Sweden). In this TPS there are two different algorithms: TMR10 (Tissue maximum ratio 10) and Convolution.

With the TMR10 algorithm, the dose calculation is done without considering the different densities of the tissues in the adjacencies of the target volume. In other words, this algorithm models all tissue in the head as water. Considering the convolution algorithm a correction of heterogeneity is applied.

Although it is possible to use both algorithms, the vast majority of users carry out the planning using the TMR 10 algorithm. Regardless of the dose calculation algorithm used, the planning aims to determine: the best position of the treatment isocenters, the size of the collimators and their respective contributions (weights) to obtain a conformed dose distribution and the lowest scattering in order to minimize the dose in organs at risk (OAR).

For treat a vestibular schwannoma is common use 12 Gy as dose treatment During the planning, the physician and physicist prescribe this dose using an isodose line. The 50% isodose line—IDL is by far the most common selection or GK based dose delivery. This is largely based on historical precedent and the assumption that prescribing to the 50% IDL provides the steepest dose fall-off outside the target. In order to obtain a dose conforming, multiple isocenters are used, especially when the shape of target volume is irregular. Isocenter associated with an irradiation geometry are called shots.

It may seem intuitive in some complex plans to place isocenter at the edge of the target volume, or even, outside the target volume. When this occurs these isocenters contribute to a dose outside the target volume, which is contrary to what is desired for a radiosurgery planning. Knowing these particularities, the vestibular schwannoma was chosen because of their significant dependence on plan conformity and selectivity [5]. This study evaluates the plans of 64 patients who underwent stereotactic radiosurgery between February 2014 and December 2018. These 64 patients correspond to all patients with diagnosis of vestibular schwannoma treated in the Gamma Knife^®^ Neurosurgery Department, Division of Oncology of Heart Hospital in São Paulo.

The evaluation of the plans for radiosurgical treatment with gamma knife® equipment is done by the physicians and the physicists using metrics quality already established. These metrics, coverage (C), selectivity (S), gradient index (GI) and conformity index (CI_paddick_). They are based on dose distributions considering the volume of the prescription isodose, the volume of the target, the isodose volume equivalent to 50% of the prescription isodose and the intersection between these volumes. This study objective was: to study the global dose distribution using the ratio (IV/TV) in a radiosurgery planning considering other isodoses volumes, larger and smaller than the prescription isodose. It is intended to evaluate the dose-failure, as well as the dose gradient within the target volume. It was tried to correlate the target volume and sphericity degree to existent indexes. This is important because with this analysis we can study if it is possible to improve the evaluation of the plans using the sphericity degree. Therefore, to develop this study having as reference the dose distribution obtained for the planning of a sphere with equivalent volume.

## II. Material and methods

### II.1 Study design and ethics

The Research Ethics Committee of Hospital do Coração (REC—HCor) approved the study under the code 3.093.147/2018/December. This study was designed as a retrospective study, focusing on the correlation between the dose distribution of vestibular schwannoma treatment plans and sphericity degree. The data of all patients were collected and analyzed anonymously.

### II.2 Study population and equipment

Sixty four patients were retrospectively identified from the gamma plan^®^ database in the Gamma Knife^®^ Neurosurgery Department, Division of Oncology of Heart Hospital in São Paulo. They correspond to all vestibular schwannoma patients treated between February 2014 and December 2018. Plans were crafted with the Leksell Gamma Plan^®^ (LGP) version 10.0 configured for the Leksell Gamma Knife^®^ Perfexion (LGKP)—(Elekta Instruments, Stockholm, Sweden). The LKGP is a standard equipment for cranial stereotactic radiosurgery (cranial SRS). The LGKP consists of 192 sealed sources of ^60^Co arranged on eight movable sectors, each sector accommodating 24 sources, and a collimator system of tungsten with aperture of 4, 8, and 16 mm. The sources move over the tungsten cone during exposures to provide circular fields of 4, 8 and 16 mm of diameter.

Each plan is performed by a physicist using a combination of forward and inverse plan methodology and the values of the metrics coverage (C), selectivity (S), gradient index (GI) and mean dose were obtained directly from LGP. For shot density, conformity index (CI) and the ratio IV/TV some numerical dose-volume histogram are exported from LGP and analyzed using the software Excel. The fourth metric “treatment time (*T*_*beam−on*_) is not evaluated because the treatments are performed with a long-time interval of up to four years, which is equivalent to almost one half-life (*T*_1/2_) of the radionuclide Cobalt-60. In Table 1 there is a resume with a description of the population.

**Table 1:** Treatment plan parameters–Vestibular Schwannoma.

Factor	Value
N° of dose plans	64
Prescription isodose (%)	
mean	51
range	45–80
N° of isocenters	
range	3–37
Shot density (number of shots / cc)	4,04–33,0
Target volume (cc)	
mean	1,85
range	0,11–8,32
Sphericity degree—ϕ	
mean	0,69
range	0,50–0,91
Coverage–C	
mean	0,98
range	0,93–1,00
Selectivity–S	
mean	0,78
range	0,54–0,93
Gradient index–GI	
mean	3,11
range	2,59–3,33
Conformity index–CI	
mean	0,81
range	0,55–0,95

The shot density values were calculated using the software Excel independently of the technique of planning.

### II.3 Treatment plans characteristics

**Fig 1:**
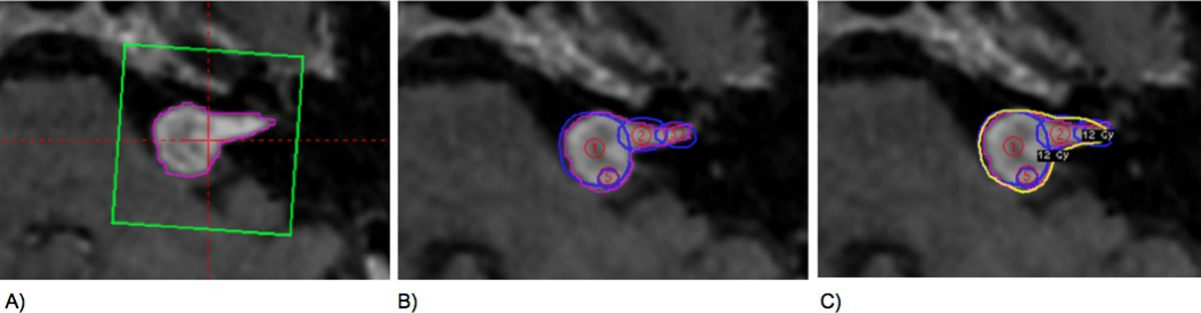
Calculation matrix, number of the isocenters and dose distribution. (A) For each target volume defined, a calculation matrix is created, which automatically encloses the volume to adjust the position and the grid size to enclose the selected target volume. (B) multiples isocenters are used, especially when the shape of target volume is irregular. (C) dose distribution of plan (yellow line is prescription isodose line = 12 Gy).

Images (DICOM data) of the study population, MR imaging, T1, T2 and FIESTA are uploaded into iplan RT^®^ Image software (version 3.0.0 BrainLAB AG, Germany). Multi-slice interpolation and automatic segmentation tools are used by the physicians to segment the organs at risk (OAR) and the target volume (TV). Each volume is then converted to a 3D structure volume and exported, in DICOM format, to Leksell Gamma Plan^®^ (LGP) software. In the LGP, the first step is to define the calculation matrix size (Fig 1A) of sufficient dimension to calculate all isodose volume (IV) and, as second step, a dose of 12 Gy is prescribed using the 50% (IDL) isodose as marginal target volume dose. In this work, all plannings are developed by a medical physicist using the TMR 10 algorithm (heterogeneity correction is not considered). It is combined forward planning tools and inverse planning settings. Therefore, the weight of each isocenter and sector settings are not uniform (Fig 1B). The dose distribution for each plan is obtained using multiples isocenters inside de target volume (Fig 1C).

### II.4 Sphericity degree (*φ*)

**Fig 2:**
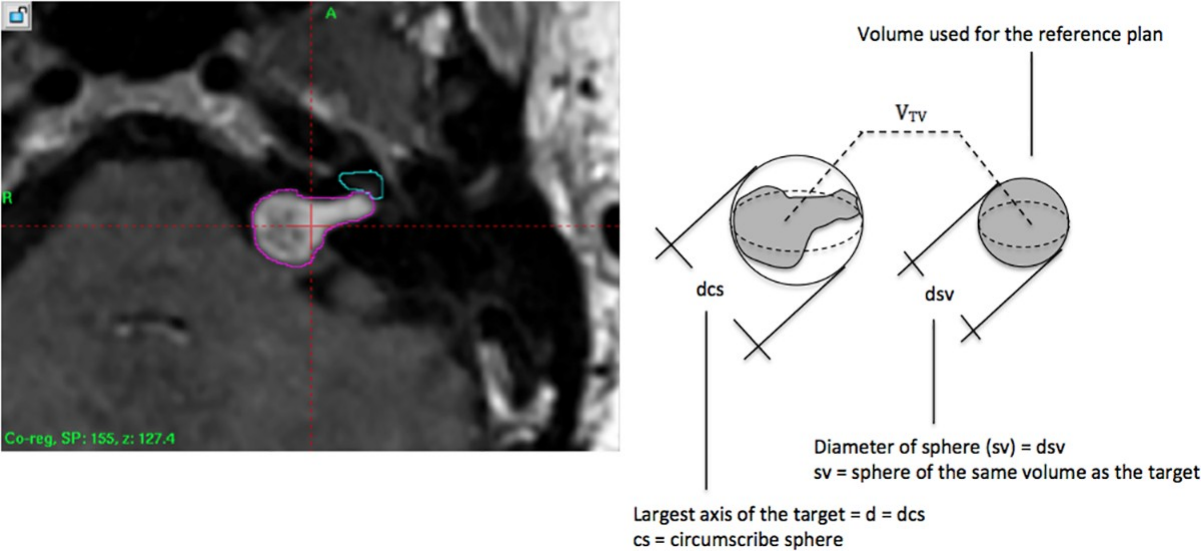
Sphericity degree of target volume. Target volume dimensions to calculate the sphericity (*φ*). Mathematics equation is defined by Wadell [13].

The concept of sphericity (*φ*) was applied to the segmented target volume. Sphericity was defined as a measurement of how closely the shape of an irregular volume (V) approaches that of a sphere. In this study (*φ*) is considered as “sphericity degree” and volume (V) as target volume (TV). The comparison of target volumes of various shapes with a sphere can be performed. It is considered surface area, volume and ratios between orthogonal axes. In its theoretical definition, operational sphericity [6] (Fig 2) is given by;

$$\varphi =\frac{V_{P}}{V_{CS}}$$ (1)

where *V*_*P*_ is the irregular target volume and *V*_*CS*_ is the volume of the smallest circumscribing sphere. This equation may be approximated by;

$$\varphi =\sqrt[{3}]{\frac{d_{SV}}{d_{CS}}}$$ (2)

where *d*_*SV*_ and *d*_*CS*_ *are the* diameter of equivalent sphere (sv) and the diameter of circumscribe sphere (cs), respectively.

The sphericity degree *φ* of the sphere is, of course, 1.00 and values of *φ* of many irregular volumes are always smaller than one,

IfTV=Sphere→φ=1

IfTV≠Sphere→φ<1

Tumor volume for each treatment plan was obtained using the dose-volume histogram (DVH) from LGP. The diameter was measured on a RM image, used for treatment planning. Considering (*φ*) values obtained for all target volumes, they are classified into six groups: Group A: 0.50 ≤ φ_TV_ ≤ 0.58; Group B: 0.61 ≤ φ_TV_ ≤ 0.66; Group C: 0.67 ≤ φ_TV_ ≤ 0.69; Group D: 0.70 ≤ φ_TV_ ≤ 0.74; Group E: 0.75 ≤ φ_TV_ ≤ 0.77; Group F: 0.78 ≤ φ_TV_ ≤ 0.91.

### II.5 Plan with equivalent spherical volume and optimization strategy

**Fig 3:**
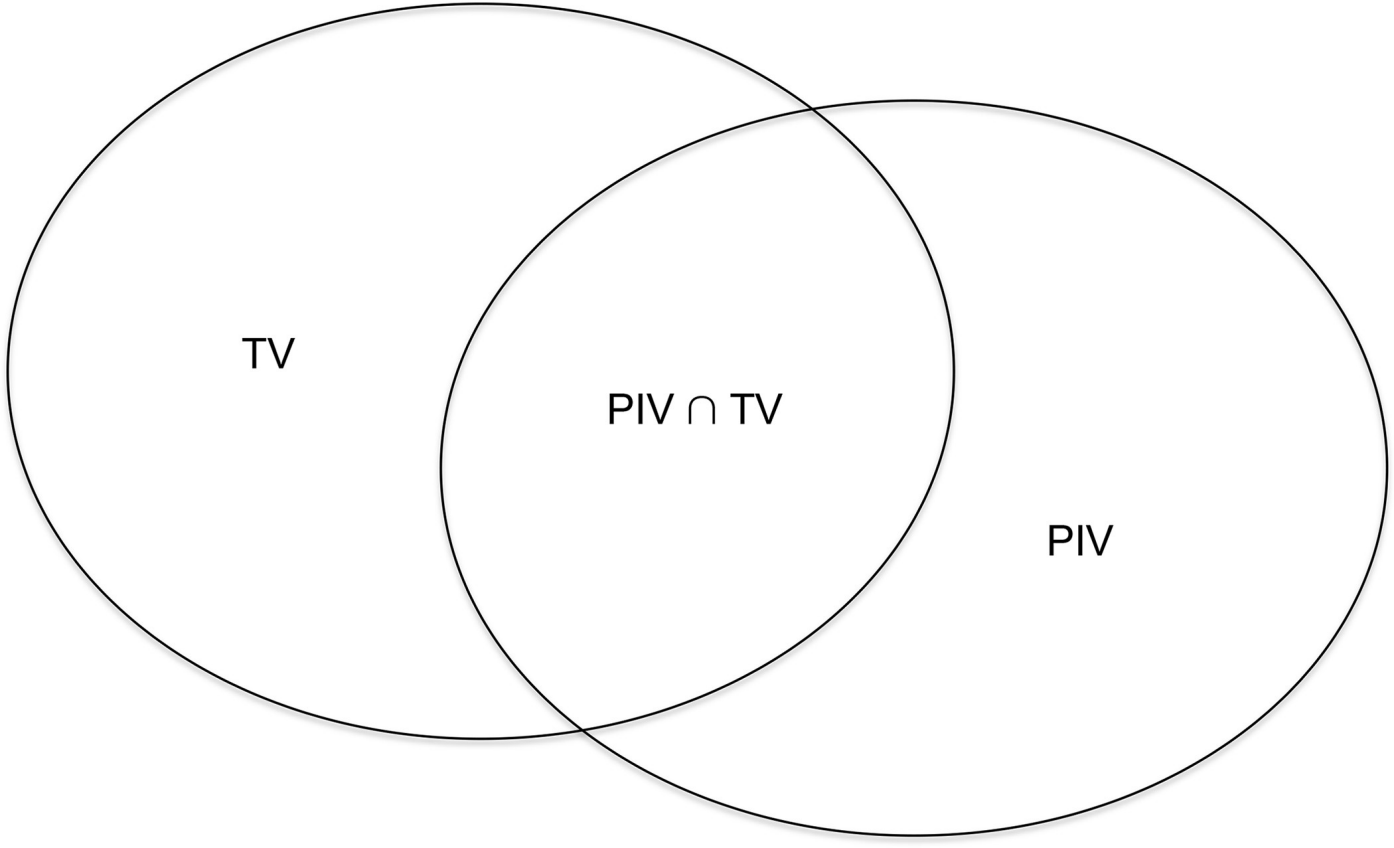
Dose metrics. Diagram to illustrate coverage, selectivity, gradient index and PIV_radius_ and equations.

The planning was made using a TPS, Leksell Gamma Plan®, version 10.1. The process to obtain this plan was divided into four steps: (a) calculate the diameter (d_eq_) of an equivalent sphere (V_sphere_ = V_TV_) to the target volume; (b) delineate this sphere within a spherical phantom; (c) define this equivalent sphere as target volume; and (d) define planning parameters;

The steps are described below:

- The calculated target volume *V*_*TV*_ was obtained for the each treatment plan, using the LGP. The sphere volume *V*_*sphere*_ and (*d*_*eq*_) of each target is calculated using the equation
  $$V_{sphere}=V_{TV}\to r_{sphere}=\sqrt[{3}]{\frac{3}{4\pi }.V_{TV}}\to d_{eq}=d=2.r_{sphere}=2.\sqrt[{3}]{\frac{3}{4\pi }.V_{TV}}$$ (3)
- The spherical phantom used in TPS, whose diameter is 160 mm, corresponds to the dosimetry calibration phantom for gamma Knife equipment. The equivalent sphere V_sphere_ is delineated as the target inside of this phantom such that it is concentric. This condition allows to affirm that the center of the sphere, as well as the center of the phantom, are positioned at the focal point of the Leksell Gamma Knife^®^ Perfexion (LGKP). This point is defined inside the stereotactic volume as (X, Y, Z = 100, 100, 100);
- To define the spherical target volume (*φ*_*TV*_ = 1), the Gamma Plan^®^ software uses this sphere as the target volume. This parameter is important to use IP settings.
- In this planning, due to the spherical geometry of both the target volume and the phantom, forward planning for small volumes was used together with combining forward planning tools and inverse planning settings for larger volumes. In the inverse planning, the following four metric entities (Fig 3) are used to calculate the treatment plan for each target volume (TV) [7].

$$Coverage=C=\frac{V(PIV\cap TV)}{V(TV)}$$ (4)

$$Selectivity=\frac{V(PIV\cup TV)}{V(PIV)}$$ (5)

$$Gradient\;Index=GI=\frac{V({PIV}_{iso/2})}{V({PIV}_{iso})}$$ (6)

$$Beam‐on\;time=T_{beam-on}=\sum_{i=1}^{N_{iso}}T_{beam-on,i}$$ (7)

**Fig 4:**
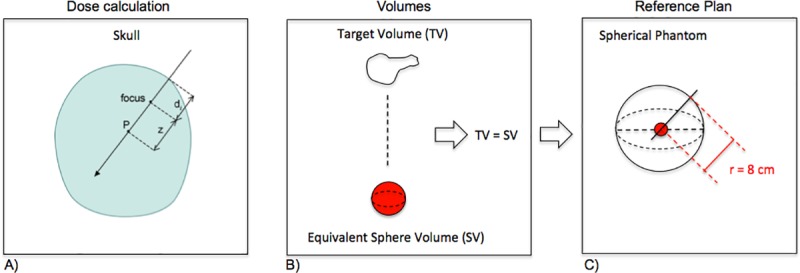
Plan with equivalent spherical volume. (A) Contribution parameters to define $\dot{D_{i}}$ using algorithm TMR 10. (B) Calculation of equivalent spherical volume for target. (C) equivalent spherical target and phantom.

The optimization is performed in two steps. First, applying a forward planning, the medical physicist defines a reasonable configuration of shot sizes and locations. Next, an inverse planning settings is used to produce a final treatment plan. The parameterization of the dose radiation is done considering: dose of 12 Gy prescribed at the 50% isodose as first option and for metrics C and S, which are complementary, we began set 0.5 and 0.5, respectively. From the above equations shown in the Fig 4 we can observe that in the calculations of C, S and GI, there is a common term, the PIV corresponding to the volume of the isodose prescription. Three-dimensional dose distribution is then calculated using the LGP. It summates the dose distribution from individually measured beam profiles. This dose is correlated by dose rate $(\dot{D_{i})}$ using the Eq 5 from TMR 10 algorithm, it doesn’t use heterogeneity correction. As previously mentioned, TMR 10 algorithm is the most used by the Gamma Knife^®^ radiosurgery centers. This is the modeling of the depth dose. The dose rate is attenuated by two terms with different attenuation parameters: in the first term, the distance from the skull surface is multiplied by *μ*_0_ = 0,00633 *mm*^−1^ [8], which is the attenuation coefficient of the primary photon fluency along the beam, and in the second term the distance from the focus point is multiplied by the virtual attenuation coefficient *μ*_*i*_ of the particular beam. It describes the contribution to dose at z due to photon–electron interactions in the neighborhood of *P*.

$${\dot{D}}_{i}={\dot{D}}_{calibration,16}.\frac{1}{192}.{of}_{i}.e^{\mu_{0}(d_{i}-R_{calibration})}.\frac{e^{\mu_{i,z}}}{{\left(1+\frac{Z}{R_{xx}}\right)}^{2}}$$ (8)

It can be observed that: in the plan considering the spherical target and phantom, this parameter $\dot{D_{i}}$ will be the same for each one beam of LGKP. The (Fig 4A–4C) is a schematic figure of plan with equivalent spherical volume.

Therefore, dose metrics are obtained from LGP software for each treatment plan and plan with equivalent spherical volume. Another parameter evaluated in this study is the mean dose; this value is obtained using DVH from LGP.

### II.6 Metrics to evaluate the plan

The metrics to evaluate the dose distribution in a radiosurgery planning are coverage (C), selectivity (S), gradient index (GI), Paddick conformity index (CI_Paddick_) and isodose volume (IV) inside and outside the target.

The Eqs (4–7) represent the calculation of C, S, GI and beam-on time respectively. The software provides these metric values at the end of the dose calculation. The GI is a powerful tool that can be used to objectively measure the dose falloff outside the target and can also be used to demonstrate the optimal prescription isodose, so that the steepest possible dose falloff for any given isocenter configuration is achieved. This metric can be used for any prescription isodose, therefore to calculate GI by software, *PIV*_*iso*_ and *PIV*_*iso*/2_ volumes are used. Reffering to CI_Paddick_, there is a proportional relationship between the coverage of the target volume $\frac{{TV}_{PIV}}{TV}$ and the proportion of the volume of the prescription isodose within the target volume $\frac{{TV}_{PIV}}{PIV}$ [9]. The CI value for each plan is calculated using the *TV*_*PIV*_ and *TV* volumes

$${CI}_{Paddick}=\frac{{TV}_{{PIV}^{2}}}{\left(TV.PIV\right)}$$ (9)

**Fig 5:**
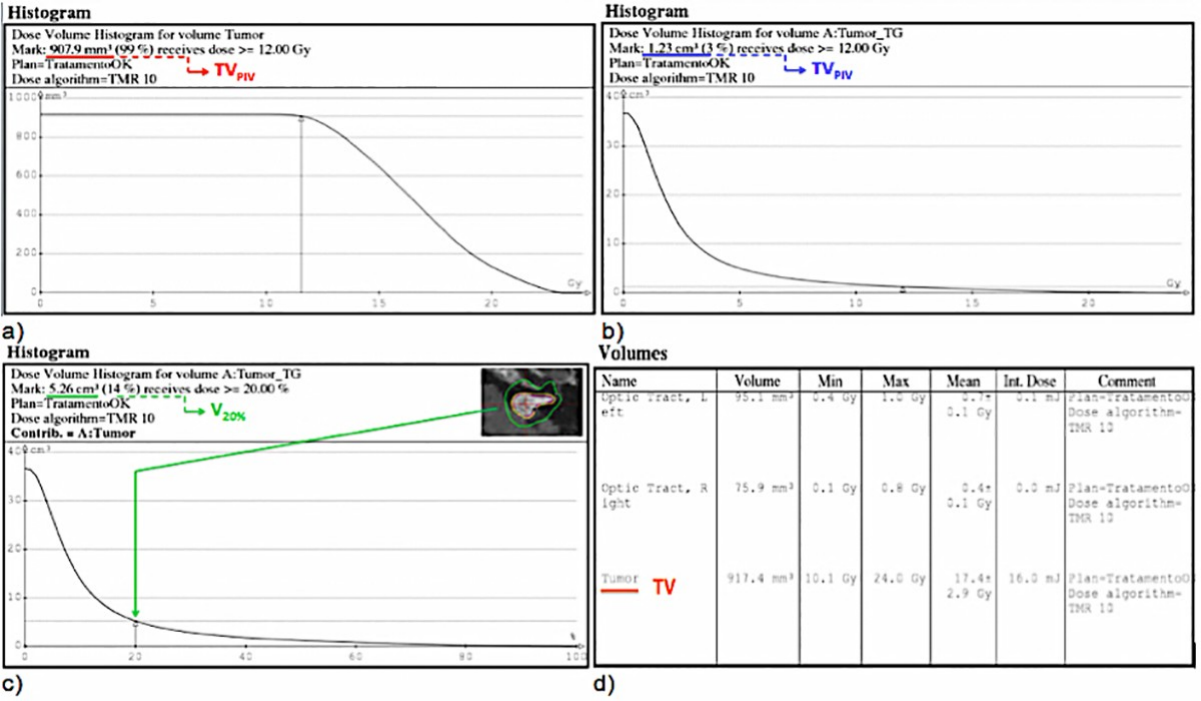
Dose-volume histogram (DVH). (A) Dose-volume histogram for Tumor. (B) Dose-volume histogram for Matrix (absolute dose). (C) Dose-volume histogram (relative dose). (D) Table volume analysis. Tools on LGP to obtain the Paddick conformity index (CI_Paddick_).

To obtain *TV*_*PIV*_, *TV*, *PIV*, *PIV*_*iso*_ and *PIV*_*iso*/2_, the dose-volume histogram (DVH) shown in (Fig 5A–5D) is used. It is obtained from the TPS (Leksell Gamma Plan^®^ software).

With the dose distribution obtained from all plans, we investigate if sphericity degree can be correlated to coverage (C), selectivity (S), gradient index (GI), Paddick conformity index (CI_Paddick_). In this study, we evaluate *φ* (considering both irregular volumes and equivalent spherical volumes). For this reason, it is verified if the correlations are present, namely:

φ∝C;φ∝S;φ∝GI;φ∝CI (10)

and considering the dose distribution

$$\varphi_{target}\propto \left[(\frac{V_{10\%}}{V_{TV}});(\frac{V_{20\%}}{V_{TV}});\ldots (\frac{{TV}_{50\%}}{V_{TV}})\ldots (\frac{V_{90\%}}{V_{TV}})\right]$$ (11)

$$\varphi_{sphere}\propto \left[(\frac{V_{10\%}}{V_{TV}});(\frac{V_{20\%}}{V_{TV}});\ldots (\frac{{TV}_{50\%}}{V_{TV}})\ldots (\frac{V_{90\%}}{V_{TV}})\right]$$ (12)

## III. Statistics

In this study an software was used based on a rigorous mathematical approach. For a better understanding of this uncertainty, it is important to separate it into four parts: (a) the uncertainty of X coordinate of a point; (b) the uncertainty of Y coordinate of a point; (c) the uncertainty of Z coordinate of a point (D) the uncertainty of determining the dose in a voxel. Therefore, the summary defines the total uncertainty associated with the measurements of this work. This value is estimated to be the half of a pixel length, because the dose point is not calculated exactly over the intersection of two voxels, but over the middle of a voxel, the program will automatically detect the border as if it were over one end of this voxel. This procedure is valid for all axes. The pixel length in the x, y and z axes are values obtained from a previous characteristic of image to planning. Therefore, we can estimate that the uncertainty value in the dose calculation in this work is half of the smallest unit of measure, which for volume is 0.05 mm^3^. Similarly, for the dose value the uncertainty is 0.05 Gy.

## IV. Results

The proposed dose distribution analysis is a correlation between the dose spreading and sphericity degree of target volume. This analysis can be used for any isodose. This study has the advantage to demonstrate that de sphericity degree (*φ*) can be related to the dose distribution (IV/TV).

### IV.1 Shot density

**Fig 6:**
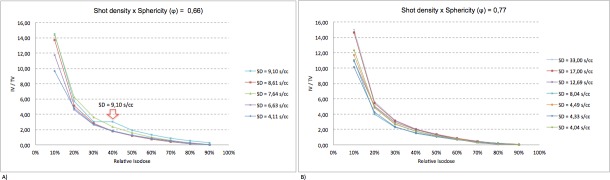
Quantitative analysis of ratio IV/TV. **(A)** Graph presenting the dose distribution against the ratio between isodose volume (IV) and target volume (TV) considering (**φ**) = 0,66 for different shot densities (SD). **(B)** Graph presenting the dose distribution against the ratio between isodose volume (IV) and target volume (TV) considering (**φ**) = 0,77 for different shot densities (SD).

In (Fig 6A and 6B) it is possible to observe the variation of the parameter shot density with the volume of the target. The range of this variation is around 4,04–33,0.

### IV.2 Correlation between sphericity degree (*φ*) and dose metrics

**Fig 7:**
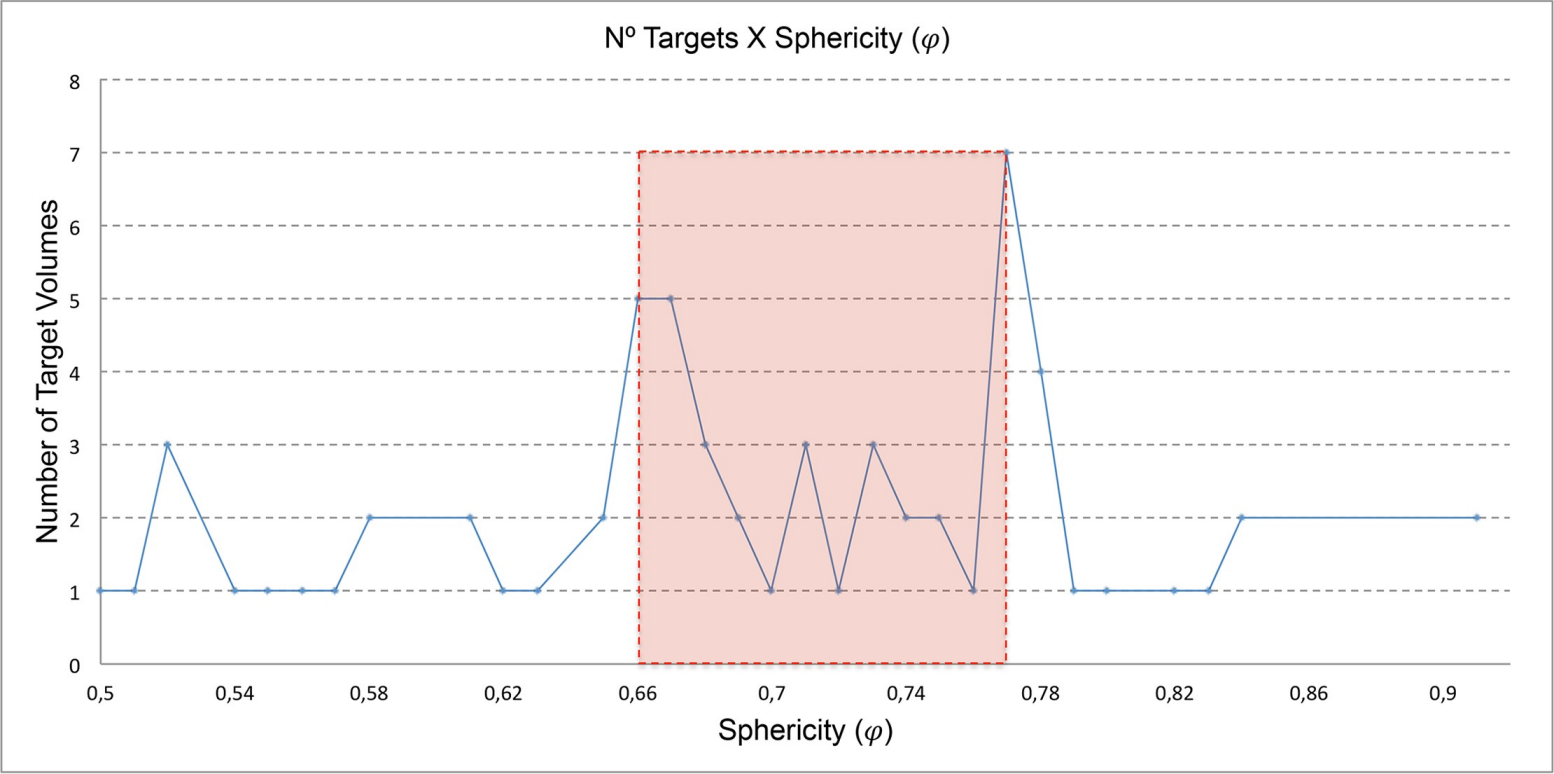
Quantitative analysis of targets and their respective sphericity degree.

In order to better evaluate if there is a proportional relationship between the sphericity degree and the metrics already used to evaluate a planning, we verify what range of the sphericity degree (ϕ) and its respective target volumes. The target volumes of all 64 patients are plotted according to sphericity degree in Fig 7.

It is possible to observe that the majority of these volumes has (ϕ) around 0,66–0,77, corresponding 54,7% of the total. These small sphericity values justify the high number of shots as smaller collimators. The use of these smaller collimators leads to a longer treatment time [10–11].

In treatment plans with sphericity 0,77, the number of the shots varied from 6 to 34 and their volumes from 0,18–5,94. The largest shot density is 33,00 s/cc. This high value of shots density does not imply differences in the dose distribution, because of the greater degree of sphericity (Fig 6B).

**Fig 8:**
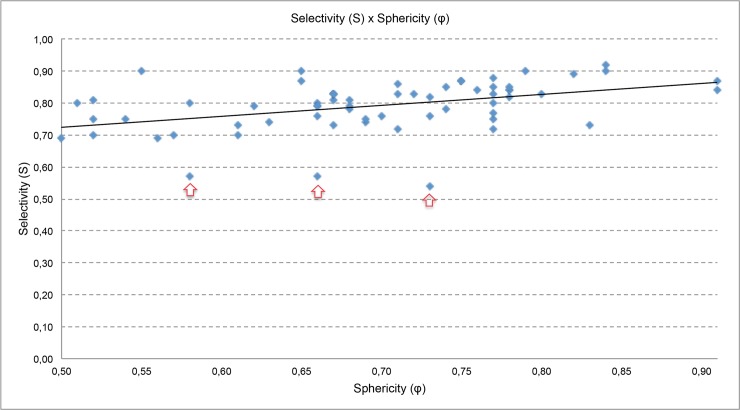
Selectivity (S) versus sphericity degree (*φ*).

The selectivity (S), fraction of prescription isodose volume (PIV) within target volume (TV), can be calculated using inverse of conformity index multiplied by coverage or using the Eq 5 (provided by software Gamma Plan®). For all plans evaluated in this study, the smaller and larger selectivity values obtained were 0,54 and 0,92, respectively. Considering this range, in Fig 8, the selectivity (S) as a function of the sphericity degree (ϕ) is plotted.

**Fig 9:**
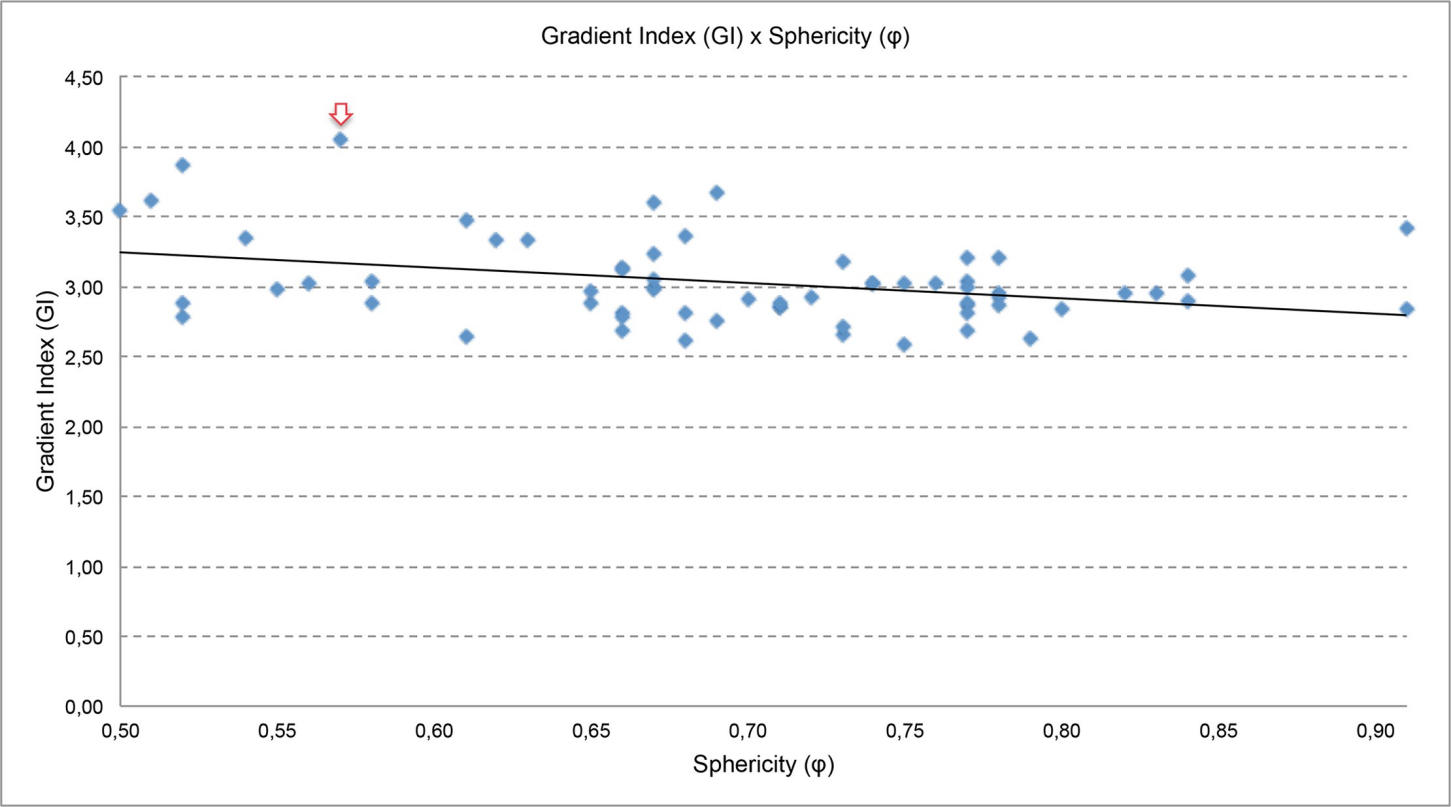
Gradient index (GI) versus sphericity degree (*φ*).

Considering the gradient index (GI) as the ratio of the volume enclosed by the half of the prescription dose to that volume enclosed by the prescription dose, the data are in Fig 9. For all plans evaluated in this study, the smaller and larger gradient index values obtained were 2,61 and 4,05, respectively. Considering this range, in Fig 9, the gradient index (GI) as a function of the sphericity degree (ϕ) is plotted.

**Fig 10:**
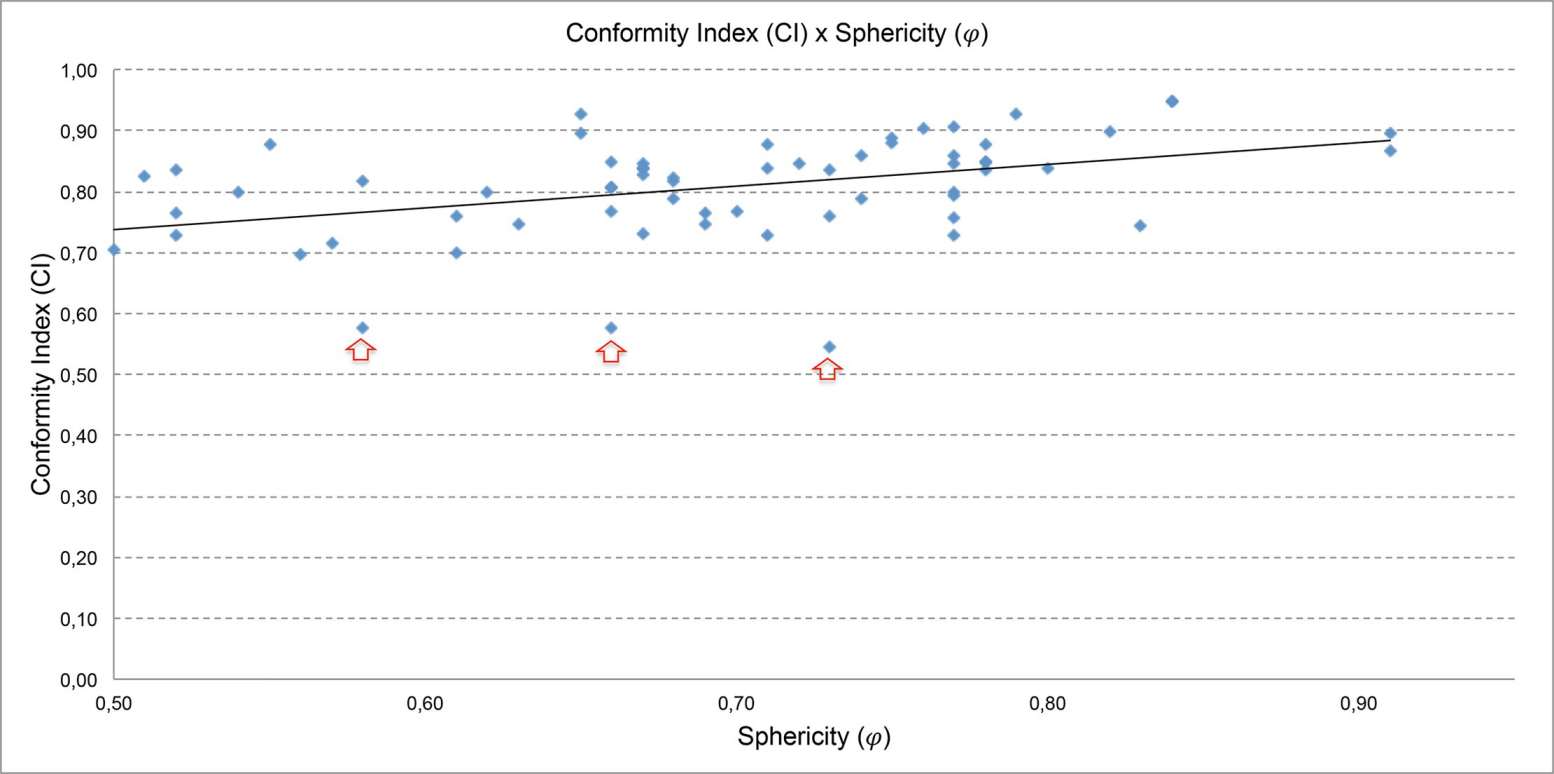
Conformity index (CI) versus sphericity degree (*φ*).

Considering the conformity index (CI), calculated using the Eq 9, the values obtained are plotted in Fig 10. It is possible to observe that: the higher the value of the sphericity, the value of the conformity index (CI) tends to be closer to 1, which is the value used as an indicator of the quality of a plan for radiosurgery.

**Fig 11:**
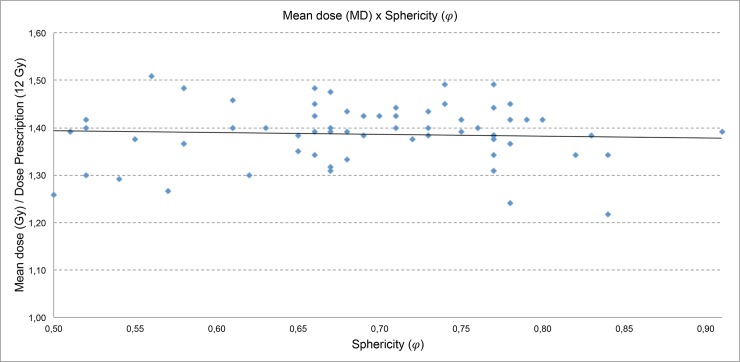
Mean dose (MD) versus sphericity degree (*φ*).

In Fig 11 the mean dose (MD) value is divided by the prescription dose value in order to better define the scale of the graph. In this graph the mean dose value varies independently of the sphericity degree (**φ**).

**Fig 12:**
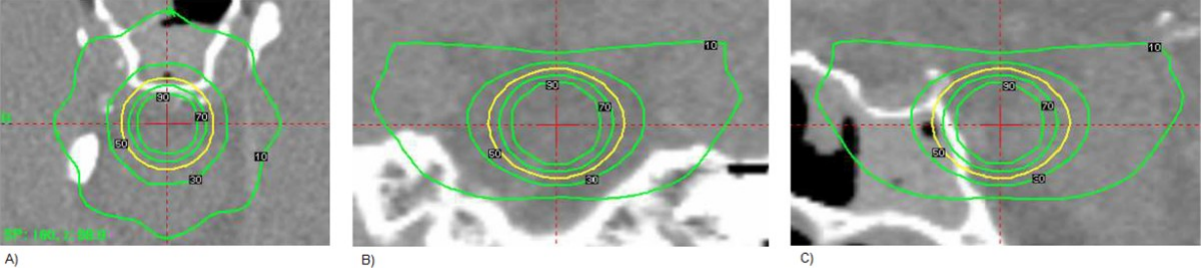
Dose distribution of a reference plan. The target volume is spherical (Yellow Line = Prescription Isodose) with a diameter of 8 mm: (a) Axial plane, (b) Coronal plane and e (c) Sagittal plane.

**Fig 13:**
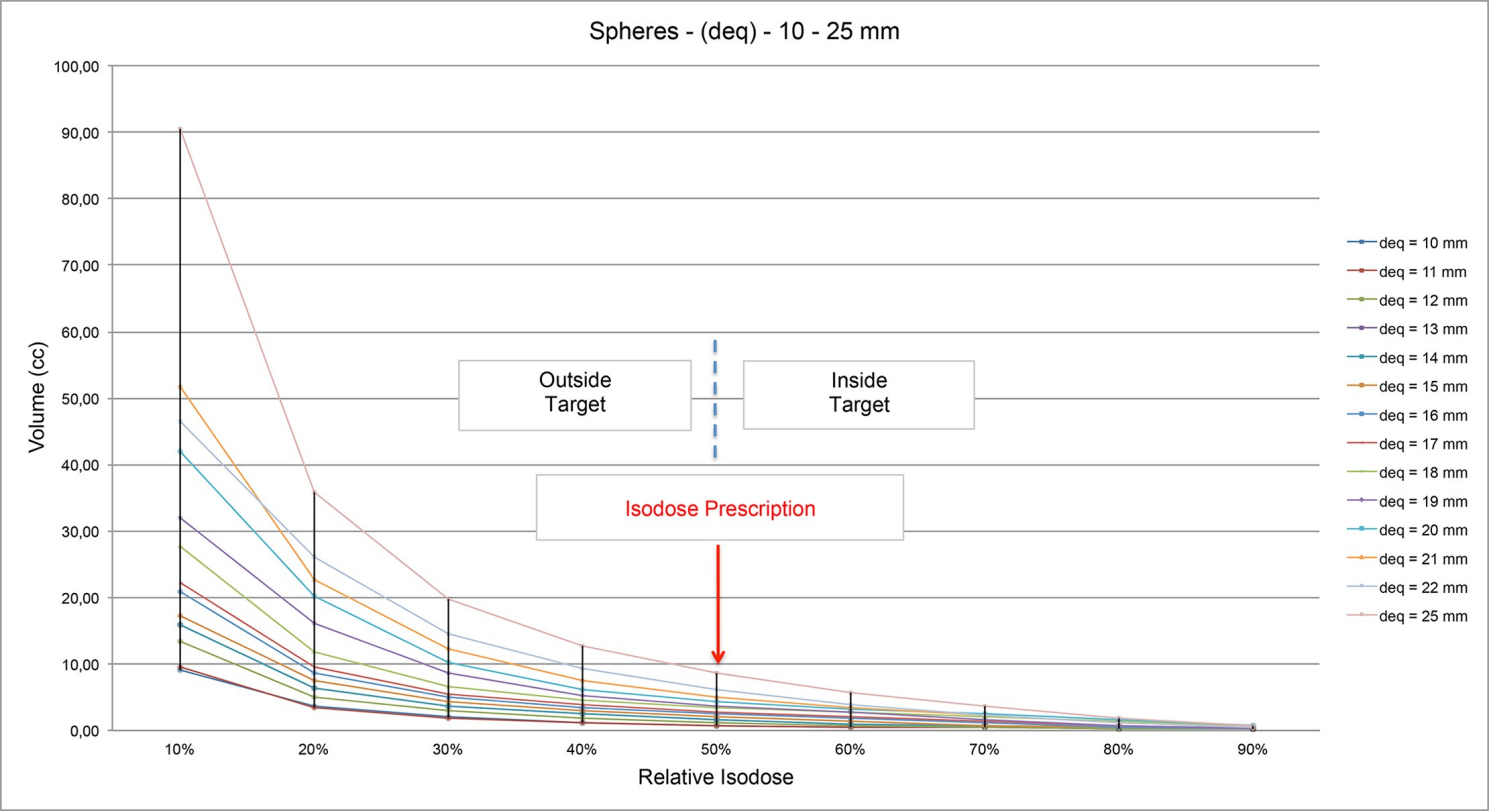
Quantitative analysis of relative isodoses volumes for different spherical target volume.

In order to evaluate the dose distribution in plans considering the target and the phantom, both spherical, in Fig 12 it is shown an example of the dose distribution of a spherical target and and in the Fig 13 is shown a quantitative analysis of relative isodoses for different spherical target volume.

**Fig 14:**
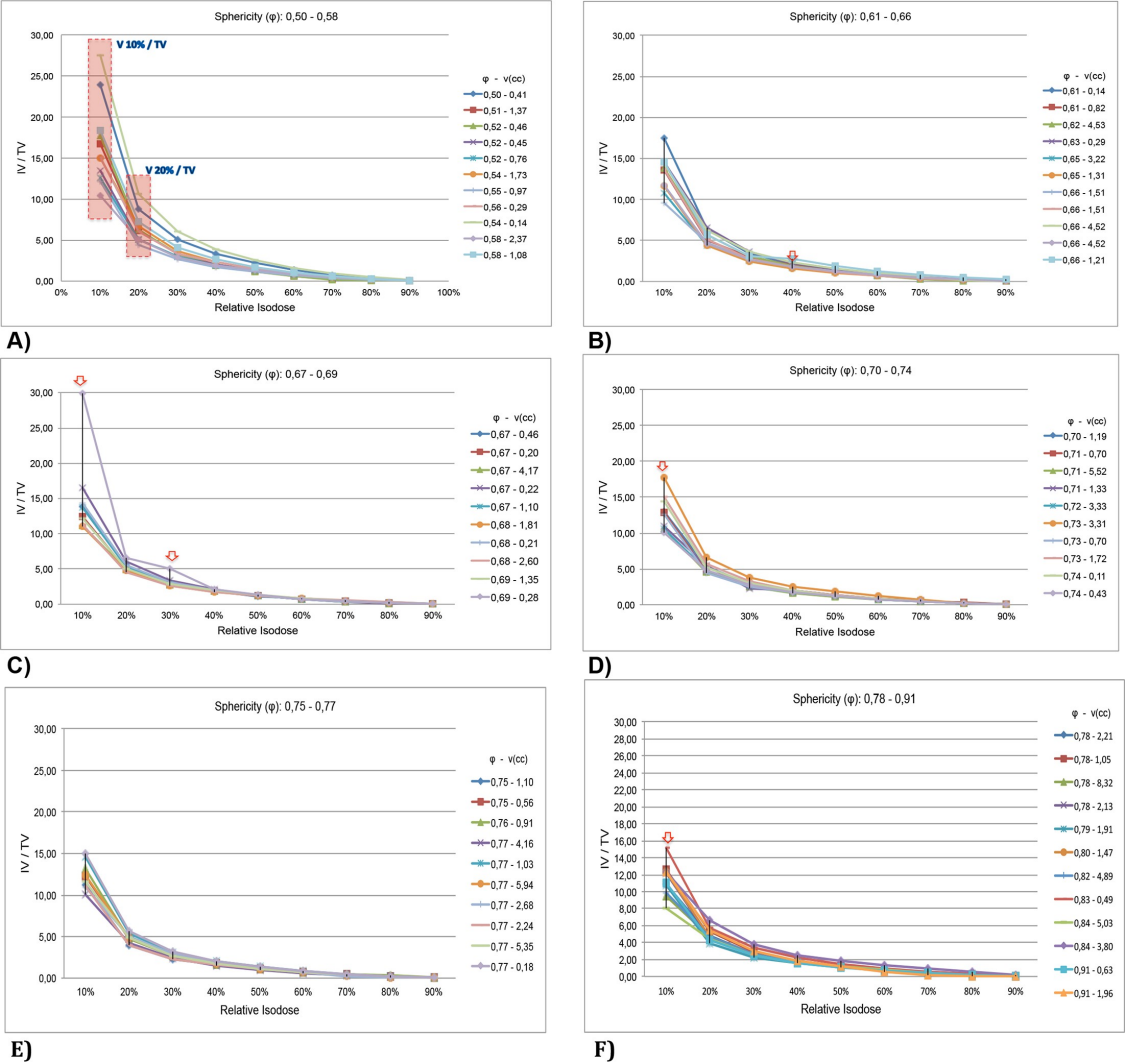
Dose distribution considering range of sphereicity degree. (A) Sphericity degree (φ: 0,50–0,58). (B) Sphericity degree (φ: 0,61–0,66). (C) Sphericity degree (φ: 0,67–0,69). (D) Sphericity degree (φ: 0,70–0,74). (E) Sphericity degree (φ: 0,75–0,77). (F) Sphericity degree (φ: 0,78–0,91).

As reported in item II.4, the sphericity degree found for all treatment plans are divided into 6 groups. The objective of this division is to be able to interpret if the ratio IV / TV for each body has the same behavior when compared to the distribution obtained in the plans whose target and phantom are spherical. In (Fig 14A–14F) evaluate the dose distribution (IV/TV) for each group.

In Fig 14A it is shown that the ratio IV/TV for low dose is higher because the sphericity degree φ is low. Specially $\left[(\frac{V_{10\%}}{V_{TV}});(\frac{V_{20\%}}{V_{TV}})\right]$. In Fig 14B it is observed that the $\left[(\frac{V_{40\%}}{V_{TV}})\right]$ is higher. It is larger than expected. Investigating this specific treatment plan we observe that the shot density is larger. For the same reason this effect occurs with 2 treatment plans shown in Fig 14C. Analogously, this effect occurs 1 treatment plan shown in Fig 14D and 14F. In the (Fig 14D–14F), the dose distribution presents the expected behavior for all treatment plans.

## V. Discussion

Our study shows that the sphericity degree offers important information about the dose distribution outside and inside the target volume. This is not evaluated by the other parameters already implemented as metric to analyze the GKP plans.

The GK plans are actually prescribed to isodose lines considering the minimum coverage of target volume. By instead characterizing the gradient as a distance from the prescription IDL, the resulting data can be used to better assess the feasibility of certain treatments. However, to evaluate de dose distribution with the implemented metrics, the gradient index is a ratio between two volumes: prescribed isodose volume and volume of 50% prescribed isodose line. For this reason the data was expanded to evaluate the dose distribution inside and outside the target volume for any isodose line. Therefore, with the dose distribution obtained from all plans, we investigate if sphericity degree can be correlated to coverage (C), selectivity (S), gradient index (GI), Paddick conformity index (CI_Paddick_). For vestibular schwannoma, however, a strong case can be made for the utilization of this methodology, because the lesions are not completely spherical.

The following information correlation between sphericity degree (φ) and dose metrics.

*Shot density (SD)*: In this this work, the shot density was characterized for all plans developed by physicist using the forward planning or inverse planning. This large variation in shots density occurred because the plans were made combining the two forward and inverse planning techniques. This analysis was made to verify if there is a dependence relationship between the sphericity degree and the shot density. As the results show, this relationship cannot be concluded because it also depends on which technique was used for planning.

*Sphericity (φ)*: The ratio IV/TV are not equals for plans with the same sphericity, because some parameters used in their respective plans are not the same. In treatment plans with sphericity 0,66, the number of the shots varied from 11–30 and their volumes from 0,88–5,11. The largest shot density is 9,10 s/cc and this can justify the difference between dose distributions in and out target (Fig 7A).

*Selectivity (S)*: In Fig 8, the obtained data were adjusted by a straight line to establish a trend line. It indicates an increase of the selectivity value when the sphericity value increases. In some plans, the selectivity is very small and evaluating these plans it is observed that these low values are due to isodose prescription lower than 50%.

*Gradient index (GI)*: The obtained data were adjusted by a straight line to establish a trend line. It is possible to observe that: the higher the value of the sphericity, the value of the Gradient Index tends to be closer to 3, which is the value used as an indicator of the quality of a plan for radiosurgery using gamma Knife^®^ equipment. Also in graph 9, it is possible to observe that in a plane the value of the gradient index is high. Evaluating this plan, it is observed that the higher GI values are related to prescription doses smaller than 50%.

*Conformity index (CI)*: As in the analysis made for the selectivity, in some plans, the conformity index is very small and evaluating these plans it is observed that these low values are due to isodose prescription lower than 50%.

*Mean dose (MD)*: Evaluating the plans, observe that the mean dose is higher for the plans with the greater number of isocenters. Therefore, although it is not possible to equate the proportionality ratio of the sphericity degree with the mean dose, we can say that the mean dose increases when the shot density (SD) increases too. The variation of the mean dose, independently of the sphericity degree (**φ**) justifies an evaluation of this parameter using the convolution algorithm to evaluate if there is a dependence heterogeneity of the tissue in this anatomical area [12–13].

Several important information can be obtained from Fig 12.: a) isodoses volumes larger than the prescription isodose present the same behavior, varying in proportion to the volume of the spherical target. b) the volumes of the isodoses smaller than the isodose of prescription also present a behavior proportional to the volume of the target, but this behavior does not prevail for the isodose of 10%. This variation can be observed in the dose distribution obtained in a planning (Fig 13). c) Still in the graph in Fig 13, we can see that the ratio of 50% isodose volume per target volume is close to 1, which shows us S and IC values close to 1. d) The variation between the ratios between IV / TV is higher for the larger spherical TVs due to the fact that although the target is spherical, for larger volumes, more shots are needed to obtain the best C, S and GI values. However, this increase in shots density implies a variation in the dose distribution, mainly outside the target volume.

There is evidence that this methodoly is acceptable considering that standard GKP radiosurgery are commonly prescribed in 50% IDL [14].

## VI. Conclusion

This study showed that when the IV / TV ratio values for different isodoses reflect the same behavior when compared with the IV / TV ratios obtained in the target volume and phantom (both spherical) schedules, the results provide information that can characterize the behavior of the dose distribution (IV / TV) according to the degree of sphericity of the target volume. However, it was not possible to equate a direct relation of the degree of sphericity with the metrics, which did not make it possible to infer an analysis of the quality of the planning considering this relation. Also in this study, it was possible to observe that for high number of shots, that is, high shots density, there exists a higher spreading of low doses.

The new proposed dose distribution analysis using the sphericity degree is simple to understand and easy to calculate. With this analysis is possible a more global evaluation of the dose gradients around the TV and dose distribution inside and outside de target volume, for a larger range of isodose lines. Therefore, it is valuable tool when an objective comparison between two or more treatment plans and to verify if is possible improve the plan considering low dose spreading.

## Supporting information

S1 File Ethics statement. Submission Satatement to Ethics Committee of Hospital do Coração.
(PDF)

S2 File Ethics statement. Ethics Statement of Committee of Hospital do Coração (REC—HCor) approved the study under the code 3.093.147/2018/December.
(PDF)

S3 File Ethics statement. Ethics committee waived the requirement for informed consent.
(PDF)

S4 File Funding statement. Funding Statement author.
(PDF)

S5 File Data sheet of Vestibular Schwannoma. This is the data sheet with all data used in this study.
(XLSX)

S6 File Term of confidentiality. Term of confidentiality of authors.
(PDF)
