# Correction: Gamma Knife radiosurgery for vestibular schwannomas: Evaluation of planning using the sphericity degree of the target volume

**DOI:** 10.1371/journal.pone.0303715

**Published:** 2024-05-09

**Authors:** Crystian Wilian Chagas Saraiva, Simone Coutinho Cardoso, Daniela Piai Groppo, Antônio Afonso Ferreira De Salles, Luiz Fernando de Ávila, Luiz Antonio Ribeiro da Rosa

In of the Materials and Methods section, there are errors in the first, second, fifth, ninth and twelfth equations. In Eqs 1 and 2, the cube root has been mistakenly inserted in the equation of the diameter (Eq 2) instead in the equation of volume (Eq 1). Please view the complete, correct Eqs 1 and 2 here:

$$\varphi =\sqrt[{3}]{\frac{V_{P}}{V_{CS}}}$$ (1)

$$\varphi =\frac{d_{SV}}{d_{CS}}$$ (2)

In equation five, a union sign appears in the numerator, but it should be an intersection sign. Please view the complete, correct equation five here:

$$Selectivity=\frac{V(PIV\cap TV)}{V(PIV)}$$ (5)

In equation nine, it is not clear that the entire numerator is squared; the numerator should be bracketed. Please view the complete, correct Eq 9 here:

$$CI=\frac{{\left({TV}_{PIV}\right)}^{2}}{TV.PIV}$$ (9)

In Eq 12, the sphericity degree symbol is included, but it should not be. Please view the complete, correct Eq 12 here:

$$Sphere=\left[\left(V_{10\%}\right);\left(V_{20\%}\right);\ldots \left(V_{50\%}\right);\ldots \left(V_{90\%}\right)\right]$$ (12)
