# Supplementary material for: Gamma Knife radiosurgery for vestibular schwannomas: Evaluation of planning using the sphericity degree of the target volume
Source: PLoS One. 2020 Jan 10;15(1):e0225638. doi: 10.1371/journal.pone.0225638 (PMC6953829; doi:10.1371/journal.pone.0225638)
Supplement: S1 File — Submission Satatement to Ethics Committee of Hospital do Coração. (PDF) [file pone.0225638.s001.pdf]

MINISTÉRIO DA SAÚDE - Conselho Nacional de Saúde - Comissão Nacional de Ética em Pesquisa – CONEP

FOLHA DE ROSTO PARA PESQUISA ENVOLVENDO SERES HUMANOS

1. Projeto de Pesquisa:
OTIMIZAÇÃO DA DISTRIBUIÇÃO DE DOSE EM PLANEJAMENTOS DE RADIOCIRURGIA REALIZADOS EM UM EQUIPAMENTO GAMMA KNIFE® PERFEXION

2. Número de Participantes da Pesquisa: 69

3. Área Temática:

4. Área do Conhecimento:
Grande Área 1. Ciências Exatas e da Terra

PESQUISADOR RESPONSÁVEL

5. Nome:
CRYSTIAN WILIAN CHAGAS SARAIVA

6. CPF:	7. Endereço (Rua, n.º):		
075.498.297-13	Rua Sampaio Viana PARAISO 180 SAO PAULO SAO PAULO 04004000		
8. Nacionalidade:	9. Telefone:	10. Outro Telefone:	11. Email:
BRASILEIRO	11988302732		crystian.saraiva@gmail.com

Termo de Compromisso: Declaro que conheço e cumprirei os requisitos da Resolução CNS 466/12 e suas complementares. Comprometo-me a utilizar os materiais e dados coletados exclusivamente para os fins previstos no protocolo e a publicar os resultados sejam eles favoráveis ou não. Aceito as responsabilidades pela condução científica do paramProjeto acima. Tenho ciência que essa folha será anexada ao paramProjeto devidamente assinada por todos os responsáveis e fará parte integrante da documentação do mesmo.

Data: 14 / 11 / 2018

Assinatura

INSTITUIÇÃO PROPONENTE

12. Nome:	13. CNPJ:	14. Unidade/Órgão:
ASSOCIACAO BENEFICENTE SIRIA	60.453.024/0003-90	
15. Telefone:	16. Outro Telefone:	
(11) 3886-4688		

Termo de Compromisso (do responsável pela instituição): Declaro que conheço e cumprirei os requisitos da Resolução CNS 466/12 e suas Complementares e como esta instituição tem condições para o desenvolvimento deste projeto, autorizo sua execução.

Responsável: ALEXANDRE BIASI CAVALCANTI CPF: 808292369-53

Cargo/Função: DIRETOR @ INST. PESQUISA HCOR

Data: 14 / 11 / 2018

Dr. Alexandre Biasi Cavalcanti
Diretor
Instituto de Pesquisa (IP) HCor

Assinatura

PATROCINADOR PRINCIPAL

Não se aplica.
