# Supplementary material for: Gamma Knife radiosurgery for vestibular schwannomas: Evaluation of planning using the sphericity degree of the target volume
Source: PLoS One. 2020 Jan 10;15(1):e0225638. doi: 10.1371/journal.pone.0225638 (PMC6953829; doi:10.1371/journal.pone.0225638)
Supplement: S2 File — Ethics Statement of Committee of Hospital do Coração (REC—HCor) approved the study under the code 3.093.147/2018/December. (PDF) [file pone.0225638.s002.pdf]

PARECER CONSUBSTANCIADO DO CEP

DADOS DO PROJETO DE PESQUISA

Título da Pesquisa: OTIMIZAÇÃO DA DISTRIBUIÇÃO DE DOSE EM PLANEJAMENTOS DE RADIOCIRURGIA REALIZADOS EM UM EQUIPAMENTO GAMMA KNIFE®

Pesquisador: CRYSTIAN WILIAN CHAGAS SARAIVA

Área Temática:

Versão: 1

CAAE: 04137118.4.0000.0060

Instituição Proponente: ASSOCIACAO BENEFICENTE SIRIA

Patrocinador Principal: Financiamento Próprio

DADOS DO PARECER

Número do Parecer: 3.093.147

Apresentação do Projeto:

Este trabalho apresenta o desenvolvimento de plugins que permitam definir a geometria do(s) isocentro(s) dentro de um volume alvo, utilizando um software livre, ImageJ, na linguagem JAVA ou PYTON, bem com a avaliação de parâmetros de planejamento dos pacientes submetidos à radiocirurgia para neurinoma do acústico, em um equipamento Gamma Knife® Perfexion. Esses parâmetros foram correlacionados com o objetivo de identificar uma métrica para categorizar os planejamentos no software Gamm Plan®.

Objetivo da Pesquisa:

Objetivo Primário:

Tendo em vista a dificuldade em categorizar um planejamento de SRS, por não se ter uma métrica para tal objetivo, o objetivo principal deste trabalho é avaliar os dados dos pacientes já tratados no GKP, identificando e correlacionando os parâmetros que possam auxiliar na otimização da distribuição de dose e desenvolver algoritmos capazes de definir a geometria do(s) isocentro(s) dentro de um volume

Objetivo Secundário:

Os objetivos específicos desse trabalho são: -Estabelecer uma metodologia de analise de dados do planejamento de SRs feito em um TPS Gamma Plan® -Avaliar quantitativamente os parâmetros de

planejamento -Correlacionar os parâmetros de planejamento para se definir métricas para avaliação do plano de tratamento -Desenvolver um algoritmo na linguagem JAVA ou PYTON para leitura dos arquivos RT Structures, das imagens de planejamento, no formato DICOM -Desenvolver um algoritmo na linguagem JAVA ou PYTON para definir a posição do(s) isocentro(s) em um volume alvo -Desenvolver um algoritmo para definir a morfometria do volume alvo.

Avaliação dos Riscos e Benefícios:

Riscos:

Este estudo não apresenta risco ao paciente, pois trata-se de uma coleta de dados em um software de planejamento computadorizado sem a identificação do paciente, somente através do diagnóstico.

Benefícios:

Com o desenvolvimento deste estudo, será possível avaliar e aplicar uma metodologia de planejamento para tratamento que permitirá uma melhor distribuição de dose em um tempo menor de tratamento. Outro benefício que se pretende alcançar com o desenvolvimento desse estudo é conseguir reduzir o tempo que antecede o tratamento após a colocação do sistema de imobilização, procedimento esse que é feito na data do tratamento.

Comentários e Considerações sobre a Pesquisa:

Projeto com relevância científica, otimização da distribuição de dose, bem como a identificação de métricas para avaliação de um planejamento no TPS Gamma

Plan®. Diante dessa possibilidade de pesquisa, o estudo de cada um dos parâmetros separadamente, bem como a avaliação correlacionando-os irão contribuir para esse objetivo.

Considerações sobre os Termos de apresentação obrigatória:

Adequadamente apresentados.

Recomendações:

1. No preenchimento da Plataforma, sugere-se adequar a quantidade de pacientes (para 69) no campo "informe número de indivíduos, abordados pessoalmente, recrutados ou que sofreram algum tipo de intervenção neste centro de pesquisa"
2. Indicar bibliografia no texto.

Conclusões ou Pendências e Lista de Inadequações:

Vide recomendações acima.

Considerações Finais a critério do CEP:

Tendo em vista a legislação vigente, lembramos que devem ser encaminhados ao CEP HCor relatórios parciais anuais referentes ao andamento do projeto de pesquisa e relatório final ao término do estudo. Qualquer modificação do projeto original deve ser apresentada a este CEP, de forma objetiva e com justificativas, para nova apreciação.

Este parecer foi elaborado baseado nos documentos abaixo relacionados:

Tipo Documento	Arquivo	Postagem	Autor	Situação
Informações Básicas do Projeto	PB_INFORMAÇÕES_BÁSICAS_DO_PROJETO_1258346.pdf	06/12/2018 09:46:28		Aceito
Folha de Rosto	FR.pdf	06/12/2018 09:46:14	CRYSTIAN WILIAN CHAGAS SARAIVA	Aceito
Outros	DeclaracaoResponsabilidade.pdf	06/12/2018 09:40:05	CRYSTIAN WILIAN CHAGAS SARAIVA	Aceito
Outros	ProjetoCEPAvaliacaoMetodologica.PDF	06/12/2018 09:31:01	CRYSTIAN WILIAN CHAGAS SARAIVA	Aceito
Outros	Cartaisencao.pdf	28/11/2018 15:35:33	CRYSTIAN WILIAN CHAGAS SARAIVA	Aceito
Declaração de Instituição e Infraestrutura	decl_infra_Crystian.pdf	28/11/2018 15:23:29	CRYSTIAN WILIAN CHAGAS SARAIVA	Aceito
Projeto Detalhado / Brochura Investigador	projetocep.docx	28/11/2018 14:51:39	CRYSTIAN WILIAN CHAGAS SARAIVA	Aceito
Outros	TermodeConfidencialidade.pdf	28/11/2018 14:44:14	CRYSTIAN WILIAN CHAGAS SARAIVA	Aceito

Situação do Parecer:

Aprovado

Necessita Apreciação da CONEP:

Não

Endereço: Rua Abrão Dib, 50 - Térreo
Bairro: Paraíso **CEP:** 04.004-030
UF: SP **Município:** SAO PAULO
Telefone: (11)3886-4688 **Fax:** (11)3886-4689 **E-mail:** etica.pesquisa@hcor.com.br

SAO PAULO, 18 de Dezembro de 2018

Assinado por:
Alberto José da Silva Duarte
(Coordenador(a))

Endereço: Rua Abrão Dib, 50 - Térreo
Bairro: Paraíso **CEP:** 04.004-030
UF: SP **Município:** SAO PAULO
Telefone: (11)3886-4688 **Fax:** (11)3886-4689 **E-mail:** etica.pesquisa@hcor.com.br
