# Supplementary material for: Gamma Knife radiosurgery for vestibular schwannomas: Evaluation of planning using the sphericity degree of the target volume
Source: PLoS One. 2020 Jan 10;15(1):e0225638. doi: 10.1371/journal.pone.0225638 (PMC6953829; doi:10.1371/journal.pone.0225638)
Supplement: S3 File — Ethics committee waived the requirement for informed consent. (PDF) [file pone.0225638.s003.pdf]

Hospital do Coração
HCor
Associação do Sanatório Sírio

São Paulo, 27/11/2018

Ilmo Sr. Coordenador
Comitê de Ética em Pesquisa do Hospital do Coração - HCor

Pesquisador: Crystian Wilian Chagas Saraiva

Assunto: Solicitação de Dispensa do Termo de Consentimento Livre e Esclarecido (TCLE)

Eu, Crystian Wilian Chagas Saraiva, Pesquisador responsável pelo Projeto de Pesquisa: "OTIMIZAÇÃO DA DISTRIBUIÇÃO DE DOSE EM PLANEJAMENTOS DE RADIOCIRURGIA REALIZADOS EM UM EQUIPAMENTO *GAMMA KNIFE® PERFEXION*" a ser conduzido no Hospital, por este termo, solicito ao Comitê de Ética desta instituição a dispensa do Termo de Consentimento Livre e Esclarecido, em razão desta pesquisa apresentar caráter retrospectivo, por tratar de levantamento de dados junto à um Sistema de Planejamento Computadorizado sem a identificação dos dados do paciente.

Comprometo-me a preservar a privacidade dos sujeitos de pesquisa, garantindo que os dados coletados serão utilizados única e exclusivamente para a execução do projeto em questão, e que as informações divulgadas, de maneira nenhuma identifiquem o sujeito de pesquisa.

Espero ter atendido satisfatoriamente às exigências desse Comitê, aguardamos retorno.

Atenciosamente,

CRYSTIAN WILIAN CHAGAS SARAIVA
Pesquisador Principal
