# Supplementary material for: Gamma Knife radiosurgery for vestibular schwannomas: Evaluation of planning using the sphericity degree of the target volume
Source: PLoS One. 2020 Jan 10;15(1):e0225638. doi: 10.1371/journal.pone.0225638 (PMC6953829; doi:10.1371/journal.pone.0225638)
Supplement: S4 File — Funding Statement author. (PDF) [file pone.0225638.s004.pdf]

To
Editorial Board of PLOS One

São Paulo 23th August, 2019

Dear Joerg Heber, M.D, Ph.D.
Editor-in-Chief

PLOS One

We are submitting the paper **"Gamma Knife radiosurgery for vestibular schwannomas: evaluation of planning using the sphericity degree of the target volume".**

There is no conflict of interest to be declared and it was developed in accordance with ethical international regulations.

All costs in this study were the responsibility of the author Crystian Wilian Chagas Saraiva, having no any cost to the institution. There was no any funding for the development of this study.

We look forward to receiving an evaluation from the editorial board.

Sinceraly,

Crystian Wilian Chagas Saraiva

Center of Radiotherapy and Radiosurgery Gamma Knife of Heart Hospital and Insitute of Radiation Protection and Dosimetry – IRD
