# Supplementary material for: Gamma Knife radiosurgery for vestibular schwannomas: Evaluation of planning using the sphericity degree of the target volume
Source: PLoS One. 2020 Jan 10;15(1):e0225638. doi: 10.1371/journal.pone.0225638 (PMC6953829; doi:10.1371/journal.pone.0225638)
Supplement: S6 File — Term of confidentiality of authors. (PDF) [file pone.0225638.s006.pdf]

Hospital do Coração
HCor
Associação do Sanatório Sírio

TERMO DE CONFIDENCIALIDADE PARA UTILIZAÇÃO DE DADOS

Título do Projeto

Referência: OTIMIZAÇÃO DA DISTRIBUIÇÃO DE DOSE EM PLANEJAMENTOS DE RADIOCIRURGIA REALIZADOS EM UM EQUIPAMENTO *GAMMA KNIFE®* *PERFEXION*

Cadastro no CEP

Os pesquisadores do presente projeto se comprometem a preservar a privacidade dos sujeitos da pesquisa, cujos dados serão coletados em um Sistema de Planejamento Computadorizado da **Unidade de Radiocirurgia – Gamma Knife.** Concordam, igualmente, que essas informações serão utilizadas única e exclusivamente para execução do presente projeto. Comprometem-se, igualmente, a fazer divulgação dessas informações coletadas somente de forma anônima.

Nome dos Pesquisadores	Assinatura
CRYSTIAN WILIAN CHAGAS SARAIVA	[signature]
LUIZ ANTONIO RIBEIRO DA ROSA	[signature]

Nome do Pesquisador Principal: CRYSTIAN WILIAN CHAGAS SARAIVA

Assinatura do Pesquisador Principal: [signature]

Local: São Paulo **Data: 19 de Novembro de 2018**
